# Retraction Note: The impact of the COVID-19 pandemic on socio-economic and sustainability

**DOI:** 10.1007/s11356-025-35926-2

**Published:** 2025-01-10

**Authors:** Xueli Wei, Lijing Li, Fan Zhang

**Affiliations:** 1 Harbin Medical University, Daqing, 163319 Heilongjiang China; 2 University of Chinese Academy of Social Sciences, Beijing, 100102 China

**Retraction Note: Environmental Science and Pollution Research (2021) 28:68251-68260**

10.1007/s11356-021-14986-0

The Editor-in-Chief and the Publisher have retracted this article. An investigation by the Publisher found a number of articles, including this one, with a number of concerns, including but not limited to compromised peer-review process, inappropriate or irrelevant references, containing nonstandard phrases or not being in scope of the journal.

Based on the investigation's findings, the Editor-in-Chief no longer has confidence in the results and conclusions of this article.

The authors have not responded to correspondence from the Publisher about this retraction.

